# Supplementary material for: Association between oral microbiome and seven types of cancers in East Asian population: a two-sample Mendelian randomization analysis
Source: Front Mol Biosci. 2023 Nov 21;10:1327893. doi: 10.3389/fmolb.2023.1327893 (PMC10702768; doi:10.3389/fmolb.2023.1327893)
Supplement: Supplementary file 1 [file DataSheet1.ZIP › Supplementary Materials/MR plots of saliva/saliva═╝/colorectal cancer/pheno.2022_to_colorectal cancer_forest.pdf]

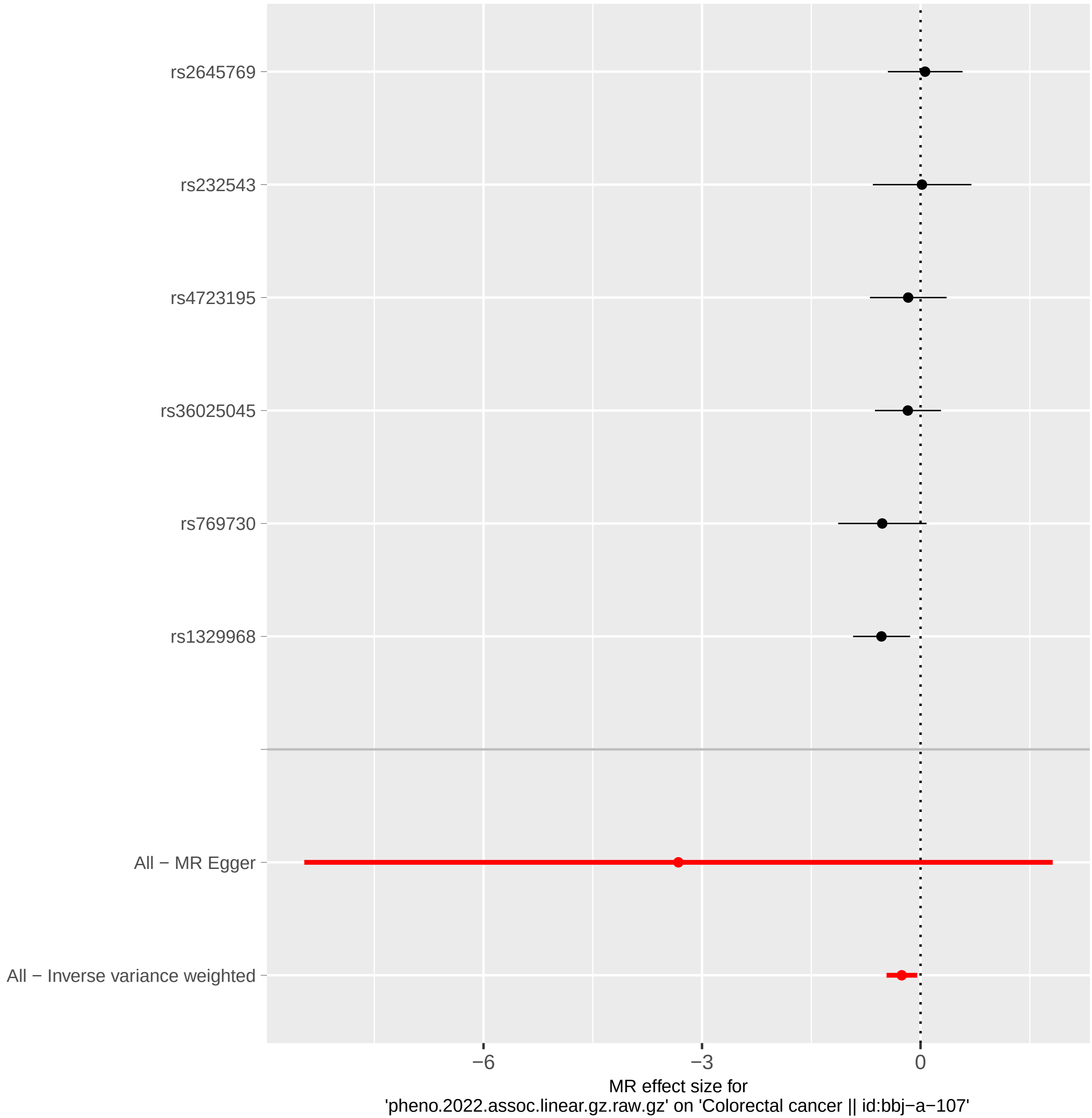

rs2645769
rs232543
rs4723195
rs36025045
rs769730
rs1329968
All − MR Egger
All − Inverse variance weighted
−6
−3
0
MR effect size for
'pheno.2022.assoc.linear.gz.raw.gz' on 'Colorectal cancer || id:bbj−a−107'
